# FACTORS AFFECTING ANHYDROBIOSIS SURVIVAL IN EUTARDIGRADE PARAMACROBIOTUS EXPERIMENTALIS (MACROBIOTIDAE)

**DOI:** 10.1093/geroni/igac059.2932

**Published:** 2022-12-20

**Authors:** Amit Kumar Nagwani

**Affiliations:** Adam Mickiewicz University, Poznan, Wielkopolskie, Poland

## Abstract

**Background:**

Tardigrades, considered among animals the most resistant to complete loss of body water (desiccation) occurring during anhydrobiosis, are also interesting because of their place between the two primary invertebrate model organisms (Caenorhabditis elegans and Drosophila melanogaster). The effect of anhydrobiosis intensification on tardigrades’ age-specific survival has rarely been studied. The eutardigrade Paramacrobiotus experimenatlis is an excellent model of the study because their remarkable capacity for anhydrobiosis. This gives also possibility to apply the species in research on anhydrobiosis as an anti-aging strategy. Aim: Main goal of present study was to determine the effect of anhydrobiosis intensification, due to application of different duration and numbers of desiccation episodes, on the age-specific survival of Pam. experimenatlis.

**Methods:**

Tardigrades were cultured under laboratory conditions, divided according to their age and sex, and subjected to several short and long desiccation episodes (3 and 30 days, respectively). The revival from desiccation was evaluated after 2-48h following rehydration.

**Results:**

Individuals` age and anhydrobiosis intensity affected tardigrades` survival. Young adult animals survived the best, and the oldest ones the worst. The number of desiccation episodes affected animal survival stronger than desiccation duration.

**Conclusion:**

The results are important for anhydrobiosis study as an-anti aging strategy. The work was supported by the research grants of the National Science Centre, Poland, NCN: 2016/21/B/NZ4/00131 and 2021/41/N/NZ3/01165.

